# Understanding EFL learners’ excessive use of translation software: An extension of the flow theory

**DOI:** 10.1371/journal.pone.0335610

**Published:** 2025-11-14

**Authors:** Shuhan Yang

**Affiliations:** School of Foreign Languages and Cultures, Panzhihua University, Panzhihua, China; Islamic Azad University Ahvaz Branch, IRANISLAMIC REPUBLIC OF

## Abstract

Grounded in flow theory and the person-artefact-task (PAT) model, this study examines the impact of flow on EFL learners’ excessive use of translation software and identifies the antecedents of flow. This study also investigates whether students’ academic background (English vs. non-English) moderates the relationship between flow and excessive use. Data from 575 Chinese university students were analyzed using Partial Least Squares (PLS) path modeling, revealing that flow significantly predicts excessive use, especially among non-English major student. In terms of the antecedents, social norms exhibit the strongest predictive power on flow, followed by task-technology fit and perceived translation quality, while task perception does not significantly influence flow. In addition, the explanatory power of the model was significant, evidenced by an R² value of 0.577 for flow and 0.272 for excessive use of translation software. These findings underscore the importance of flow in understanding excessive use behaviors and inform educational strategies that promote balanced technology use.

## 1. Introduction

Once criticized as a “bad model”, machine translation has been transformed by neural machine translation and large language models into a “good model” [1] characterized by increasing precision and accessibility. This shift has fueled extensive adoption of translation software among English as a Foreign Language (EFL) learners, significantly reshaping learning behaviors [2]. This trend aligns with early observations that even low-proficiency second language (L2) learners actively use translation tools to navigate language learning challenges [3], underscoring such software’s integral role in contemporary EFL education.

Extant research confirms that translation tools support EFL learners cognitively, affectively, and metacognitively [4–6]. By providing scaffolding and immediate feedback, they reduce anxiety [7], enhance interaction [1] and improve language proficiency [2]. This has spurred efforts to integrate translation software as a useful pedagogical tool in EFL classroom [7,8]. However, concerns persist about excessive use [3,5,9], as overdependence may diminish critical thinking, reduce comprehension of complex texts, and hinder learners’ ability to think and express in English [10]. Overdependence may also impede bilingual and intercultural competence, core aspects of language proficiency [11]. These competing perspectives raise a critical question: has excessive use become a tangible issue or concerns overstated? This tension underscores the need to investigate the existence and extent of excessive use.

Excessive use is often defined as reliance accompanied by negative outcomes such as impaired self-regulation and declining performance [12]. Given its detrimental consequences, substantial research has focused on students’ technology addiction in internet [12,13] and social media [14,15], while attempting to uncover the psychological mechanism underlying these behaviors [12,16]. However, EFL research has predominantly focused on effectiveness and pedagogy in translation tool use [1,17,18], with limited attention to overreliance and its psychological drivers. Against this backdrop, this study applies flow theory, a framework explaining immersive engagement with technology, to explore whether flow fosters EFL learners’ excessive use of translation software.

Flow, describing optimal experience of immersion, and positive affect with technology-related products and services [19], have been observed in EFL learners’ translation technology use [20,21]. This echoes findings in educational technology more broadly [22,23]. However, technology-mediated flow can also induce distraction and overinvolvement [24,25]. For instance, Google Translate, while generating excitement and supporting immediate task completion, may prioritize immediate completion over deliberate language practice [11]. This behavior risks encouraging academic dishonesty and impairing intercultural communication skills. Such risks suggest flow triggered by translation tools might heighten EFL learners’ overreliance. Furthermore, this effect may vary by language proficiency level, as disparities in tool usage and learning outcomes across different levels have been fully substantiated [9,10].

In conclusion, excessive use of translation software among EFL learners is an underexplored yet significant issue. Few studies have empirically examined its existence or its relationship with flow, and even fewer have identified the key antecedents of flow. Accordingly, this study attempts to fill the gap by addressing the following research questions:

**RQ1:** Do EFL learners use translation software excessively?

**RQ2:** Does flow contribute to the excessive use of translation software among EFL learners?

**RQ3:** What are the key predictors of EFL learners’ flow in translation software use?

**RQ4:** Does EFL learners’ academic background (English major vs. non-English major) moderate the effect of flow on excessive use of translation software?

This paper proceeds as follows: Section 2 reviews the literature and formulates hypotheses. Section 3 outlines the data and research methods, and Section 4 presents the research results. Section 5 discusses the results, implications and limitations, while Section 6 concludes with the major findings and contributions.

## 2. Literature background and hypotheses development

### 2.1 Flow theory

Csikszentmihalyi (1975) introduced flow theory to describe a psychological state of total immersion and enjoyment in an activity [26]. In flow, individuals feel in control and completely concentrate on the activities without being aware of themselves and noticing time passing. They feel intrinsically motivated and enjoy being reengaged in the activities [19]. They also pursue personal growth by striving to improve performance and achieve success [27].

Flow theory’s core tenets have informed research in technology-assisted language learning, showing the role of flow in enhancing learners’ intrinsic motivation [28], engagement [29] and language competence [22]. Educators are thus advised to promote flow by providing clear goals, timely feedback and adaptive challenges tailored to students’ skills and thus sustain satisfaction and tool use [30]. With advances in translation technology, research has examined EFL learners’ attitudes and emotions [5,31]. Findings indicate that most hold positive views and report experiencing enjoyable moments during interaction with such tools. Specifically, Y. Wang et al. (2024) explored whether flow influences translation postgraduates’ willingness to use these tools [21]. These studies highlight flow’s role in positive engagement. However, it remains unclear whether such emotions contribute to the overuse of translation software. After all, evidence from other digital contexts suggests the pursuit of flow can drive technology overuse [32–34]. This concern is salient because translation software use, despite its’ benefits, may lead EFL learners to avoid target language use [35] or uncritically accept machine output [4]. However, no empirical study has tested the link between flow and overuse in this setting. To address this gap, the present study draws on flow theory to investigate EFL learners’ flow in translation software use and its potential role in excessive reliance.

### 2.2 Person-artefact-task (PAT) model

The nine characteristics of flow are typically divided into antecedents (challenge-skill balance, clear goals, and unambiguous feedback), experience (concentration, merging of action and awareness, sense of control, loss of self-consciousness, and transformation of time), and consequences (autotelic experience) [19,24]. Research on computer-assisted foreign language learning has therefore focused on flow antecedents. For example, R. Li et al. (2021) found that challenge-skill balance, clear goals and feedback positively predicted Chinese students’ flow in digital English learning [36]. In translation contexts, Y. Wang et al. (2024) found perceived ease of use and perceived usefulness positively influenced translation postgraduates’ flow [21]. These findings underscore the intricate interplay of tools, cognition, and affect in technology-assisted learning. However, few studies have probed flow antecedents through dynamic interactions among users, tools and tasks in such environments. This gap motivates the present study’s use of the PAT model, which foregrounds such interactions in explaining flow.

Building on flow theory, the PAT model was proposed by Finneran and Zhang (2003) as a more comprehensive framework for elucidating flow antecedents in computer-mediated environments [19]. Unlike earlier flow models that conflated antecedent factors, this model distinguishes tasks from artefacts and posits that flow originates from dynamic interactions among users, tasks and technologies. The PAT model has been extensively applied in human-computer interaction research, including information behavior, virtual reality and gaming, website design and education. For instance, Mitre-Ortiz et al. (2023) applied the model to assess flow in virtual reality through hand motions, examining the interactions between user characteristics, task demands and technology features [37]. Similarly, Huang et al. (2025) used it to explore how interactions between students, visual programming tools and tasks influence flow, self-efficacy and sustained learning willingness in visual programming instruction [38].

**Fig 1:**
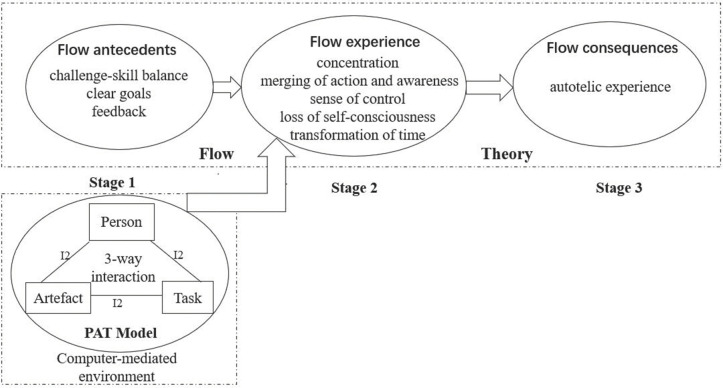
Integrated PAT-Flow framework for technology-mediated context. Note: The framework is adapted from “Stages of flow and the person–artefact–task model of flow antecedents” by Finneran and Zhang (2003). I2 stands for 2-way interaction between the components.

Fig 1 shows the linkage between flow theory and the PAT model. The PAT model emphasizes clear distinctions between persons, artefacts, and tasks, while highlighting their interactions in computer-mediated environments. In this study, these interactions replace flow theory’s key antecedents in translation software use.

### 2.3 Research model and hypotheses

**Fig 2:**
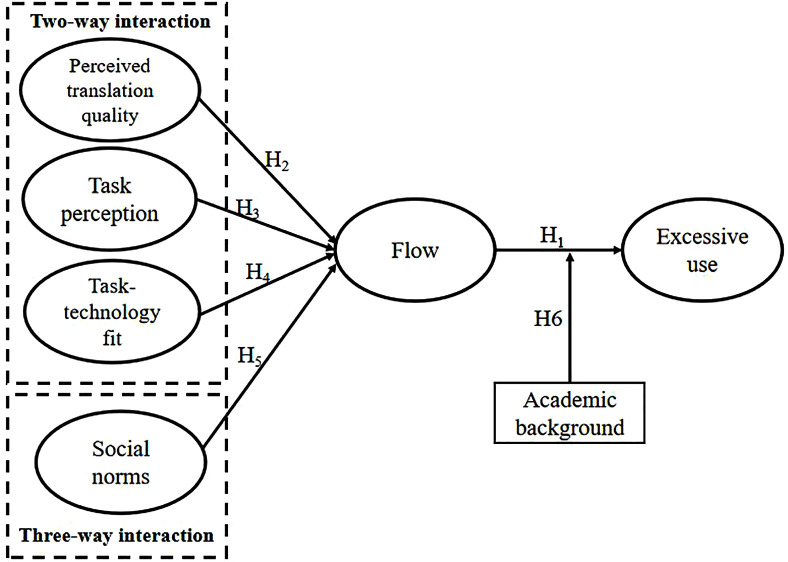
Research model and hypothesis.

Integrating flow theory with the PAT model, this study proposes a framework comprising four independent variables, one mediator, one dependent variable, one moderator, and six hypotheses (see Fig 2). Flow theory underpins the link between flow and excessive use, which suggests that flow, as a strong intrinsic motivator, may generate overreliance. The PAT model, in turn, informs the antecedents of flow by highlighting interactions among EFL learners, translation software and translation tasks. Specifically, this study examines three two-way interactions (perceived translation quality, task perception and task-technology fit) and one three-way interaction (social norms).

#### 2.3.1 Flow.

Flow refers to one’s mental state of intense focus and involvement; however, its measurement varies by context and task. In computer-mediated learning, concentration, enjoyment and sense of control are common indicators [38]. For EFL learners, translation software promotes concentration through its user-friendly interface and immediate feedback [9], while successful task completion evokes enjoyment and accomplishment [39]. Moreover, X. Wang (2021) showed that proficient use of translation technology enhances users’ sense of control over the process [40]. Accordingly, this study adopts concentration, enjoyment and sense of control as the principal dimensions of flow.

Regarding flow’s effect on translation software use, Rico & González Pastor (2022) noted translation instructors’ worries that high comfort with translation tools may encourage students’ overuse in post-editing [41]. Similarly, studies have shown that the immediate gratification provided by translation tools often leads EFL learners, especially those with low proficiency, to over-rely on translation tools despite instructors’ advice [3,42]. However, Y. Wang et al. (2024) found that flow experiences didn’t influence Chinese translation postgraduates’ intention to use such technology [21]. These inconsistencies indicate that the nature of flow’s effects on educational technology use remain underexplored. Accordingly, this study proposes the following hypothesis:

**H**_**1**_: Flow positively predicts EFL learners’ excessive use of translation software.

#### 2.3.2 Perceived translation quality.

In the PAT model, person-artefact interaction refers to users’ perceptions of tool features that influence flow [19]. Research on technology-mediated communication has widely recognized perceived ease of use as a key manifestation of this interaction [19,38], as it reflects the balance between users’ perceived skills and technical challenges posed by the tool. When this balance is achieved, users can avoid technological anxiety and obtain optimal experience of using technology. Nevertheless, modern translation tools such as Youdao Translate, Baidu Translate, Google Translate and DeepL are already highly user-friendly, with their intuitive interfaces, online/offline translation capabilities, and support of a variety of file formats making ease of use less distinctive. Instead, this study posits that users’ perception of translation quality, specifically perceived accuracy and contextual appropriateness of the outputs, as a key determinant of flow. Prior studies support this view. For instance, Tsai (2019) found that machine translation’s lexical accuracy enhances users’ concentration on target language [43]. Conversely, Lee (2019) noted that perceived inaccuracies in lexical, grammatical, or discourse aspects reduce EFL learners’ effortless immersion, disrupt writing fluency and reliability, thereby hindering occurrence of flow [7]. Moreover, a recent experimental study found that translation quality across AI translation, machine translation, and traditional translation approaches correlates directly with students’ emotions such as excitement, anxiety, and curiosity [5]. Thus, in the PAT model, translation quality captures the essence of person-artefact interaction by implicitly comparing outputs with users’ own skills. Accordingly, the following hypothesis is proposed:

**H**_**2**_: Perceived translation quality is positively related to EFL learners’ flow of translation software use.

#### 2.3.3 Task perception.

Many flow characteristics stem from person-task interaction, reflecting user’s perception of balance between their skills and task challenges [19]. Csikszentmihalyi (1975) indicated flow is most likely when task challenges meet or slightly exceed an individual’s skill level [26]. Subsequent studies have confirmed appropriately calibrated tasks can motivate leaners to engage, leading to deep concentration and immersion [23,44]. Achieving this balance also strengthens confidence in one’s competence and enhances perceptions of enjoyment and control [44].

Though how EFL learners’ perception of the challenge-skill balance in their task completion with translation tools impacts their flow hasn’t been empirically validated, ample studies on translation support their positive link. For instance, Mirlohi et al. (2011) found that the challenge-skill balance in different genres significantly influenced Iranian English majors’ flow in translation tasks [20]. Kruk & Kałużna (2025) reported that learners using AI translation tools or machine translation often exhibit diminished engagement, because they perceive such tools as reducing task challenges far below their skill levels, disrupting the equilibrium necessary for sustained immersion [5]. Conversely, lower-proficiency learners hold more positive attitudes toward translation software and are more comfortable with such tools in their writing tasks [3,7]. As analyzed by Sasaki et al. (2024), these tools reduce the challenges of writing directly in English for learners with limited linguistic sources, aligning task difficulty with their current skills [1]. Based on existing literature, this study posits that EFL learners’ task perception, that is, the balance between their skills and challenges of the task, positively affects their flow when using translation software. Hence, the hypothesis is formulated as follows:

**H_3_:** EFL learners’ task perception positively affects their flow when using translation software to finish translation assignments.

#### 2.3.4 Task-technology fit.

Task-technology fit, the match between an artefact’s functions and task demands, is another factor influencing flow [19]. In Information Systems studies, it refers to how well technology capabilities meet task requirements. Their fit suggests that individuals should select technology that aligns with the task requirements to maximize the value of technology and produce the required output [45]. Studies show such fit fosters flow. For instance, L-C Huang et al. (2017) found that the match between e-book store functions and searching tasks positively affected e-book users’ flow, which subsequently improved satisfaction [46]. This finding is further supported by research examining robo-advisors, which demonstrated that the fit is positively correlated with user’s flow, satisfaction and continuance intention [47].

In this study, task-technology fit is the degree to which translation software functions meet students’ demands in completing translation tasks. Currently, translation technology integrates electronic dictionaries, corpora, and online search engines, supporting requirements of different translation stages [18]. Their specific functions help enhance engagement. For instance, Garcia & Pena (2011) found post-editing functions of machine translation improve learners’ focus [3], while Lee (2019) observed that vocabulary selection and grammar correction deepen EFL learners’ immersion [7]. Moreover, feedback features of translation software provide immediate and personalized support, which not only improves translation quality but also reduces students’ anxiety [1]. These findings suggest that perceived fit between software functions and task demands promotes engagement and immersion. However, direct empirical evidence for this link remains limited. The following hypothesis is thus presented:

**H**_**4**_: Task-technology fit positively affects EFL learners’ flow when using translation software to finish translation assignments.

#### 2.3.5 Social norms.

Beyond dyadic interactions between person, artefact and task, their triadic interaction also positively influences flow [19]. This triadic interaction essentially represents the social environment where flow is triggered, with social norms (group-held beliefs or expectations guiding individual’s behavior) being an essential part [21]. While there is consensus that individuals are more likely to feel a sense of belonging and acceptance and thus a positive psychological state when their behaviors align with the expectations of their social group or community [48], social norms vary across cultures. For instance, Markus & Kitayama (1991) argued that individuals from collectivistic (interdependent) cultures such as China are more likely to have their emotions and behaviors shaped by others’ expectations and socially shared standards, as these cultures emphasize balance and connectedness with others [49]. Their perspective finds support in numerous cross-cultural studies on flow [50,51]. For instance, Liu W et al. (2015) found that flow among Chinese college athletes were less pronounced than among their American counterparts [50]. They attributed the differences to the fact that Chinese students’ emotions and behaviors are closely tied to the achievement of social group goals and adherence to collective expectations.

As noted, individuals in collectivistic cultures are highly responsive to social norms, which shape their emotions and behaviors. This makes it valuable to explore how such norms influence Chinese EFL learners’ flow in translation software use. Specifically, Y. Wang et al. (2024) offered relevant insight, noting that social norms affect postgraduates’ intention to use translation technology [21]. Their research suggests norms may also shape user experience. Yet social norms around translation software use remain divided. While its pedagogical effectiveness is acknowledged [9,10], concerns about plagiarism and overdependence persist [11]. This divergence leads to the following hypothesis:

**H**_**5**_: Social norms positively affect EFL learners’ flow of finishing translation assignments with translation software.

#### 2.3.6 Moderating effect of academic background.

The relationship between flow and other variables is heterogeneous, with its strength and direction often moderated by individual traits [52]. Regarding translation software use, studies have shown that factors such as users’ L2 proficiency, beliefs, and digital literacy significantly influence their usage behaviors [10]. Specifically, Peng et al. (2024) revealed that learners’ recognition of the machine translation quality in vocabulary, semantics and translation style, directly affect their willingness to correct output texts [53]. Such attitudinal differences, as Lee (2023) indicated, are closely linked to individuals’ language proficiency and learning goals [9].

Notably, English majors and non-English majors exhibit systematic differences across these core dimensions [54,55]. The former demonstrate higher L2 fluency, tend to have integrative goals and put in proactive effort in English learning. The latter, who often treat English as an auxiliary skill, rely more on instrumental motivation and generally show weaker L2 proficiency. As discussed earlier, inconsistent findings regarding translation software-assisted English writing among EFL learners with different language proficiency levels [3,7,8] suggest that their baseline language competency may alter how they engage with translation tools. This study therefore presents the subsequent hypothesis:

**H**_**6**_: The effect of flow on the excessive use of translation software is moderated by students’ academic background, namely English majors vs. non-English majors.

## 3. Research method

### 3.1 Questionnaire and constructs

The study employed a two-part questionnaire survey. The first section gathered basic information, including participants’ English proficiency levels and such translation software usage patterns as frequency, purpose, and reasons. The second section featured a Likert scale comprising 22 items, scored from 5 (strongly agree) to 1 (strongly disagree). These items, adapted from established scales in prior research, were tailored to assess the six constructs within the research model.

In this study, perceived translation quality denotes students’ perception of the output generated by translation software. The construct was measured using four items that assessed the accuracy and contextual appropriateness of the software’s output [56]. Task perception reflects students’ understanding about the balance between task demands and their competencies. Three items were adapted from the research of Guo et al. (2016), which were designed to measure online learners’ perception of tasks in online courses [44]. Task-technology fit signifies the congruence between the capabilities of translation software and the specific needs of students for completing translation assignments. Four items were adapted from a study pertaining to MOOCs and online learning [57]. Social norms in this study are to capture students’ reactions to collective beliefs regarding translation software use. This construct was measured with three items initially designed to measure peer influence and external influence on students’ use of ChatGPT [58].

Flow is often conceptualized as an individual’s optimal mental state during deep engagement in an activity [21,47]. As noted, computer-mediated learning studies emphasize concentration, enjoyment, and sense of control as its core components [23,44]. Accordingly, this study treats flow as a reflective construct and assesses it using four items to comprehensively represent each component. Specifically, three items from Cheng (2021) were adapted to measure concentration and enjoyment [47]. Concentration refers to the degree of focus students exhibit while completing translation tasks with the software, while enjoyment captures the pleasure students experience when using such tools. Perceived control, which reflects students’ self-assessed ability to complete translation tasks successfully, is measured using an item from the scale developed by Webster et al. (1993) [25].

Excessive use of translation software is characterized by high-frequency engagement with such tools in translation tasks and lack of control over usage. Furthermore, individuals who overuse translation software may experience psychological repercussions, such as depression and anxiety [59]. Accordingly, this study measured excessive use via two aspects, with use frequency reported in the first section and the scale focusing on attempts to control usage and anxiety experienced in the absence of such software. Four items were adapted from Chen et al. (2017) [60] and Hipp et al. (2023) [59].

The questionnaire was translated from English to Chinese, with both versions subjected to a peer review by two translation scholars to ensure linguistic accuracy, appropriateness and conciseness. Subsequently, the survey’s readability was assessed by four university students. Their feedback was carefully considered, leading to revisions of the questionnaire. With the Ethics Committee’s approval, a pilot study was initiated with a sample of 20 English majors and 30 non-English majors. The gathered data were analyzed to ascertain the constructs’ reliability and the scale’s overall validity.

### 3.2 Data collection

This study employed convenience sampling within a stratified framework to investigate Chinese undergraduates’ translation software use, a population exhibiting high-frequency engagement with such technologies for academic tasks. Aligned with exploratory research objectives requiring expedited access to target cohorts [61], this approach is validated in prior educational technology studies [2,62]. To address representational limitations, dual stratification by geography (eastern/central/western China) and institutional tier (key/ordinary universities) was implemented, with one key and one ordinary university per region selected based on administrative feasibility, totaling six universities. This design captured socioeconomic and pedagogical heterogeneity to enhance contextual validity [18].

Ethical approval was obtained from the Institutional Human Research Ethics Committee (Approval No. HRECA24−001), mandating written authorization from institutional coordinators and digital informed consent from all participants, wherein respondents affirmed voluntary participation, anonymity guarantees, and data usage protocols prior to accessing the survey. Data collection via Wenjuanxing (July 20–August 10, 2024) yielded 575 valid responses, exceeding minimum sample size from G*Power 3.1.9 analysis (d = 0.50, power = 80%, α = 0.05; n = 128) and PLS-SEM’s 10-times rule (n = 40) [63]. Table 1 confirms regional balance (eastern: 34.4%, central: 29.8%, western: 35.8%) and institutional proportionality (key: 48.3%, ordinary: 51.7%). However, overrepresentation of females (63.5%), freshmen (55.7%), and English majors (37.0%) necessitates cohort-specific interpretation.

**Table 1:** Demographic characteristics of the sample (N = 575).

Characteristics	Category	n	%
Region	Eastern	198	34.4
	Central	171	29.8
	Western	206	35.8
Institutional tier	Key	278	48
	Ordinary	297	52
Gender	Male	210	36.5
	Female	365	63.5
	Freshman	320	55.7
Grade	Sophomore	124	21.6
	Junior	81	14.1
	Senior	50	8.7
Major	English major	213	37
	Non-English major	362	63
English proficiency	Passed College English Test 6 (CET-6)	120	20.9
	Not passed CET-6	455	79.1

## 4. Analysis and results

The primary method for data analysis employed in this study was Partial Least Squares Structural Equation Modeling (PLS-SEM), utilizing the SmartPLS 4 software.

### 4.1 Common method bias

Since the data were self-reported, this study employed program control and statistical control to address common method bias [64]. For program control, questionnaires were completed anonymously. For statistical control, the PLS-based approach used by Liang et al. (2007) was employed [65]. A common method factor was added to the model and all principal construct items loaded on it. Variance of each item explained by the common method factor, single factors, confirmatory factors and principal constructs were calculated. Results showed that the method factor explained an average of 0.003 variance, while principal constructs explained an average of 0.719. The ratio of substantive average variance to method-based variance is about 239:1. Besides, all principal construct loadings were significant at the 0.001 level, while most of the method factor loadings were insignificant. These results indicate no serious common method bias in this study.

### 4.2 Measurement model

Initially, the internal consistency of the measurement model was measured using Cronbach’s alpha value and composite reliability (CR). Table 2 shows that all of the Cronbach’s alpha and CR coefficients were above 0.7, suggesting good internal consistency. Moreover, the item loadings and their average variance extracted (AVE) values were examined to test the instrument validity. As shown in the table, the loadings of all items were greater than 0.7, and all AVE values were higher than 0.5, indicating good convergent validity.

**Table 2:** Scale properties.

Variable	Item	Standard loading	VIF	Cronbach’s alpha	CR	AVE
**PTQ**	PTQ1	0.847	2.105			
PTQ	PTQ2	0.857	2.155	0.861	0.866	0.706
	PTQ3	0.862	2.258			
	PTQ4	0.794	1.841			
	TP1	0.839	1.758			
TP	TP2	0.915	2.869	0.86	0.863	0.783
	TP3	0.899	2.704			
	TTF1	0.823	1.999			
TTF	TTF2	0.871	2.370	0.879	0.881	0.735
	TTF3	0.874	2.454			
	TTF4	0.859	2.284			
	SN1	0.792	1.469			
SN	SN2	0.835	1.644	0.772	0.777	0.687
	SN3	0.858	1.703			
	FL1	0.814	1.827			
FL	FL2	0.871	2.431	0.858	0.859	0.703
	FL3	0.873	2.419			
	FL4	0.792	1.699			
	EU1	0.833	2.000			
EU	EU2	0.865	2.247	0.864	0.869	0.711
	EU3	0.864	2.206			
	EU4	0.809	1.875			

To ensure discriminate validity, the Fornell-Larcker criterion [66] was used for assessment. As shown in Table 3, the square root of each variable’s AVE in this study was higher than the correlation coefficient between any two variables, indicating the latent variables had favorable discriminant validity.

**Table 3:** Variable correlations and square roots of the AVE.

	EU	FL	PTQ	SN	TP	TTF
EU	**0.843**					
FL	0.521***	**0.838**				
PTQ	0.389***	0.537***	**0.84**			
SN	0.459***	0.705***	0.565***	**0.829**		
TP	0.212***	0.468***	0.504***	0.532***	**0.885**	
TTF	0.448***	0.687***	0.593***	0.702***	0.501***	**0.857**

Note: *** p < 0.001; Diagonal values in bold are square roots of AVEs.

VIF values were calculated to assess multicollinearity issues. Table 2 shows VIF values range from 1.469 to 2.869, with all below the threshold value of 5 [67]. It suggests that the measures of the constructs are less likely to have multicollinearity concerns.

The model fitness was evaluated and the results showed that all fit indices (SRMR = 0.053; d_ULS = 0.698; d_G = 0.331; Chi-Square = 312.974; NFI = 0.854) meet the threshold criteria proposed by Kline (2015) [68]. This indicates that the model is fit to data.

### 4.3 Structural model

The structural model was assessed using collinearity (VIF), path coefficients (β), determination coefficients (R^2^), effect sizes (f^2^), and predictive relevance (Q^2^).

In Table 4, all VIF values for the structural model are below the threshold of 5. This indicates no critical collinearity issues within the structural model.

**Table 4:** Inner variance inflation factor (VIF) values.

	EU	FL	PTQ	SN	TP	TTF
**EU**						
**FL**	1.000					
**PTQ**		1.754				
**SN**		2.235				
**TP**		1.548				
**TTF**		2.256				

**Fig 3:**
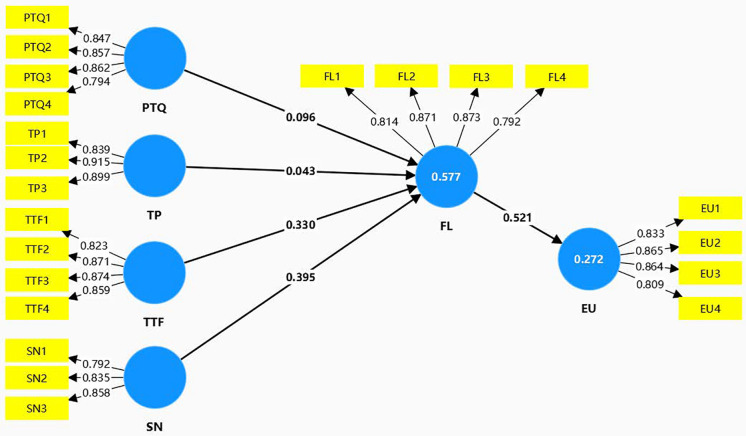
Structural model.

Table 5 and Fig 3 show the outcomes of hypothesis testing via 5000 bootstrap resamples. The results demonstrates that flow significantly affected the excessive use of translation software (β = 0.521, p < 0.001), which supported H1. The effects of perceived translation quality were positively significant at the level of 0.05 (β = 0.096), and the effects of task-technology fit (β = 0.330) and social norms (β = 0.395) were positively significant at the level of 0.001; p < 0.001). However, task perception was not positively related to flow (β = 0.043, p > 0.05). Hence, H2, H4 and H5 were supported, while H3 was rejected.

**Table 5:** Hypothesis testing results.

Hypothesis	Path	β	S.E.	T statistics	f^2^	Supported
H1	FL → EU	0.521***	0.045	11.689	0.373	Yes
H2	PTQ → FL	0.096*	0.046	2.082	0.013	Yes
H3	TP → FL	0.043	0.044	0.989	0.003	No
H4	TTF → FL	0.33***	0.052	6.347	0.114	Yes
H5	SN → FL	0.395***	0.049	8.019	0.165	Yes

Note: *** indicates p < 0.001, * indicates p < 0.05.

R^2^ reflects the explanatory power of endogenous constructs for endogenous ones. As shown in Table 6, the model explained 57.7% of the variance in flow and 27.2% of the variance in excessive use of translation software. These values exceed the medium effect benchmark for variance explanation in L2 acquisition research [69]. Regarding effect sizes (f^2^) for structural model paths, the criteria on translation technology use among EFL learners of Zhao et al. (2025) was adopted with small (0.02), medium (0.15), and large (0.35) [2]. Results showed notable variability across paths (Table 5). The path from flow to excessive use yielded an f^2^ of 0.373, meeting the criterion for a large effect and indicating a robust influence of flow on excessive use within this context. Among paths predicting flow, social norms (f^2^ = 0.165) met the medium effect criterion, task-technology fit (f^2^ = 0.114) fell slightly below this threshold, while perceived translation quality (f^2 ^= 0.013) and task perception (f^2 ^= 0.003) were below the small effect cutoff. Table 6 demonstrates Q^2^ values for flow and excessive use are above 0, indicating that the PLS-path model has predictive relevance for both endogenous constructs.

**Table 6:** Endogenous constructs assessment (R^2^ and Q^2^).

Endogenous construct	R^2^	Adjusted R^2^	Q^2^
Flow	0.577	0.574	0.40
Excessive use	0.272	0.270	0.189

### 4.4 Multiplegroup analysis

To examine whether academic background moderates the effect of flow on excessive use, this study conducted multiple analysis in SmartPLS among English and non-English majors. With guidance from Cheah et al. (2020), rigorous MICOM procedures were implemented to evaluate measurement invariance [70]. Configurational invariance was first assessed to ensure the basic structure of the measurement model was consistent across both groups. Then, compositional invariance was verified by checking that composite scores were comparable, as shown by original correlations near 1.000 for relevant constructs. Finally, equality of means and variances were evaluated. As shown in Table 7, flow met both criteria, but excessive use only satisfied mean equality. Thus, partial measurement invariance was established, allowing standardized path coefficients to be compared across groups.

**Table 7:** Results of invariance measurement testing using permutation.

Constructs	Configurational invariance (Step 1)	Compositional invariance (Step 2)		Partial measurement invariance	Equal mean assessment (Step 3a)			Equal variance assessment (Step 3b)			Full measurement invariance
		Original correlation	5%		Original differences	Confidence interval	Equal	Original differences	Confidence interval	Equal	
Flow	Yes	1.000	0.999	Yes	0.136	[-0.146,0.139]	Yes	−0.151	[-0.239,0.234]	Yes	Yes
Excessiveuse	Yes	0.999	1.000	Yes	0.017	[-0.144,0.153]	Yes	−0.274	[-0.25,0.227]	No	No

After confirming partial measurement invariance, multigroup analysis was performed using Henseler’s MGA and the permutation test. Table 8 shows the results. For the path from flow to excessive use, the coefficient for English majors was 0.299, while that for non-English majors was 0.629. Henseler’s MGA yielded a p-value of 0.001 for the difference in path coefficients, and the permutation test returned a p-value of 0.000. Both values indicate significant differences between the two groups. These results support the hypothesis that academic background moderates the relationship between flow and excessive use of translation software, with flow exerting a stronger predictive effect on excessive use among non-English majors than among English majors.

**Table 8:** Results of multigroup analysis.

Path	English majors			Non-English majors			Path coefficient differences	*p*-Value Henseler’s MGA	*p*-Value Permutation Test	Supported
	Path Coefficient	CI (95%)	*p* value	Path Coefficient	CI (95%)	*p* value				
FL → EU	0.299	[0.118,0.453]	0.001	0.629	[0.525,0.708]	0.000	0.330	0.001	0.000	Yes

## 5. Discussion

Concern has mounted over the excessive use of translation software among EFL learners. This study first examines the extent of EFL learners’ use of translation software and whether it meets the excess threshold. It then explores its association with flow through flow theory, which highlights the psychological underpinnings. Guided by the PAT model, it further scrutinizes flow’s key predictors to systematically account for EFL learners’ engagement with translation tools.

### 5.1 Flow and excessive use of translation software

This study assessed EFL learners’ use of translation software via frequency of engagement and difficulty in self-regulation. Results indicate signs of excessive use. 359 students (62.4%) reported frequent or consistent use in translation tasks, and 152 students (26.4%) adopted translation outputs without revision. In the meanwhile, the mean self-regulation score (Mean = 2.675) reflected below-average control. Although previous research highlights translation software’s effectiveness in EFL learning [7,8] and learners’ positive perceptions [4], these do not ensure responsible use. The combination of unreflective adoption and poor self-regulation reveals a gap between perceived value and actual behavior. This aligns with teachers’ concerns about over-dependence but has rarely been empirically quantified [3,41]. Moreover, findings also suggests that high-frequency use is problematic not inherently but when paired with inadequate monitoring, emphasizing the need for interventions that promote ethical and effective use, such as integrating instruction on critical evaluation of translation outputs into curricula [17,53].

Regarding the relationship between flow and excessive use of translation software, three critical observations emerge. First, this study found that flow significantly predict students’ excessive use of translation software (β = 0.521). While flow is typically regarded as a positive state associated with positive consequences, prior research has highlighted its dual nature in digital environments [71]. Studies on online gaming, social media, and smartphone use suggest that flow, by fostering enjoyment and immersion, can reduce self-regulation and encourage prolonged engagement, thereby contributing to excessive use [14,33]. A similar mechanism may operate in the EFL context, where translation software resolves immediate linguistic challenges and provides feelings of accomplishment and control. These rewarding experiences can reinforce reliance on the software, gradually substituting for the deeper cognitive processing needed for language development [17,42]. Moreover, compared with postgraduate translation students who receive systematic training in translation technologies and develop critical awareness of their limitations [18], EFL undergraduates often lack such guidance. For R.299). negative consequences. them, flow tends to emerge primarily from effortless linguistic solutions, making them more vulnerable to dependence. This contextual difference may also explain why Y. Wang et al. (2024) found no evidence that flow influences translation masters’ intention to use translation software [21]. Thus, while flow offers motivational benefits, it may simultaneously foster overreliance on translation technologies, underscoring its paradoxical role in technology-mediated learning.

Second, the explanatory power of flow in this study (R^2^ = 0.272) is more limited compared with findings in smartphone research, where flow explained 48.1% of the variance in overuse [60]. This discrepancy reflects the greater complexity of technology use in EFL learning, where reliance is influenced by multiple interacting factors such as motivation, self-regulation, pedagogical guidance, and the availability of alternative tools [9]. Prior studies have shown that variables including technology self-efficacy, technology distrust, and academic literacy enhancement all influence tool dependence. Ducar & Schocket (2018) further emphasized learners with higher metacognitive awareness may recognize when flow masks dependency, while access to supplementary tools such as dictionaries or grammar checkers may buffer against overuse of full translation software [72]. These insights suggest that interventions should not focus solely on regulating flow itself, but rather on fostering a broader ecosystem of support that integrates motivational, cognitive, and pedagogical dimensions to ensure balanced and critical engagement with translation technologies.

Moreover, non-English majors show a stronger link between flow and excessive use (β = 0.629) than their English major peers (β = 0.299). This divergence mirrors finding with translation postgraduates [21] and highlights proficiency as a moderator. According to several studies [3,7], translation software offers greater support to low-proficiency learners by directly compensating for fundamental linguistic barriers such as vocabulary and grammar, thereby reducing their anxiety and disengagement. For non-English majors, this tool-mediated resolution artificially creates a low-threshold balance between task challenges and skills, triggering flow characterized by immediate success and immersion. Yet this state induces a fragile form of flow, one sustained only through continued reliance on the tool, which predisposes these learners to overuse. In contrast, high-proficiency learners such as English majors often approach translation outputs with greater critical awareness. Rather than uncritically accepting the machine’s solutions, they actively engage in error detection and meaning negotiation, transforming tool use into an opportunity for deeper cognitive processing tend to engage differently with machine translation outputs [43,73]. As a result, their flow derives from active cognitive engagement rather than passive dependence, thereby weakening the predictive effect of flow on excessive use.

Overall, these findings suggest that in translation-technology-assisted EFL learning, flow, despite being an optimal positive state, can foster superficial reliance on the tool rather than deeper learning when pedagogical guidance and critical awareness are absent. This paradox underscores the importance of instructional strategies that leverage flow’s motivational benefits while mitigating risks of overdependence, particularly among lower-proficiency learners.

### 5.2 Antecedents of flow

Grounded in the interactive effects of the PAT model, this study proposes perceived translation quality, task perception, task-technology fit, and social norms as potential determinants of flow in the context of using translation software. Their empirical associations with flow are analyzed below.

Perceived translation quality significantly influences flow (β = 0.096), indicating students’ confidence in accuracy and contextual appropriateness of outputs enhances their likelihood of entering a state of flow. This result parallels findings with Chinese translation postgraduates, which showed that positive evaluations of translation tools significantly predict flow (β = 0.15) [21]. Both findings indicate a consistent yet modest association. Such evidence aligns with research on translation software-assisted writing, where students’ sense of control and positive emotions mainly derive from trust in the accuracy of translations, particularly grammatical and vocabulary accuracy [9,10].

Interestingly, task perception, signifying students’ perceived challenge-skill balance, showed no significant effect on flow. This contrasts with Mirlohi et al. (2011), who found such balance in different text genres influenced Iranian English majors’ flow in translation without technology [20]. Flow theory posits flow emerges when challenge matches or slightly exceeds skill. However, the integration of translation software may disrupt this balance, especially when learners adopt translated outputs directly without skill-task alignment. This is consistent with the viewpoint that Chinese university students often rate machine translation outputs as higher than their own work, reducing their motivation to adjust skills to meet task demands [43]. Additionally, Chinese students tend to prefer low challenge, high skill scenarios [51], whereas the measurement items emphasize balance between the two. This mismatch may further weaken the link between task perception and flow. Thus, how trust in translation software reshapes the effect of challenge-skill dynamics on flow needs further exploration.

Moreover, task-technology fit significantly affects flow (β = 0.330), indicating students who perceive translation software functions as meeting task demands are more likely to experience flow. This aligns with prior studies on e-book stores and robo-advisors [46,47], which found that functional alignment between technology and tasks fosters flow. In this study, the software’s capabilities meets task requirements, providing tools that strengthened perceived control and sustained focus. Survey data showed that 62.6% of participants used the software to verify translations, 66.4% to check synonyms, antonyms and alternative words, and 39.1% to review grammar structure. These data highlight its ability to meet diverse needs, reducing disruptions and enabling immersion in technology-mediated contexts.

Furthermore, social norms emerged as the strongest predictor of flow (β = 0.395). This finding extends the research of Y. Wang et al. (2024) by demonstrating that social norms directly drive flow, beyond their established influence on tool adoption [21]. Another cross-cultural study found that social norms exerted differing impacts on AI acceptance and usage between Polish and Egyptian university students [58]. Such findings indicate that flow antecedents are culturally contingent. For instance, a research found, unlike American athletes, whose flow is more tied to autotelic traits, Chinese athletes prioritize collective expectations and show less emphasis on individual challenge-seeking [50]. In this study, Chinese students’ flow was strongly influenced by teachers (Mean = 2.53) and media cues (Mean = 2.59), likely reflecting the impact of external ideas on translation software use. Accordingly, the “social norms-based flow model” identified in this study contrasts with Western “autotelic model”, where individual task engagement dominates [51].

These findings suggest translation software design should consider learners, tools and tasks, and cultural factors. Given their strong impact on student behavior and experience, social norms must be integrated into design, particularly in collectivistic contexts where educational and cultural expectations shape use norms.

### 5.3 Implications

Theoretically, this study extends flow theory by examining its paradoxical role in translation-technology-assisted EFL learning. While flow is generally conceptualized as an optimal, positive state of engagement, the findings show that it can also predict excessive reliance on translation software, thereby challenging the traditional assumption of flow as inherently beneficial. By demonstrating how enjoyment and perceived control may reinforce habitual reliance, this study advances the theoretical understanding of flow as a dual-faceted construct in educational technology use. Moreover, applying the PAT model to the EFL translation context enriches its explanatory scope. By integrating perceived translation quality, task perception, task-technology fit and social norms, this study highlights how these factors collectively shape flow, and in turn contribute to overdependence. Notably, incorporating social norms underscores the significance of cultural and social conditions, illustrating that flow is not only a cognitive-affective state but also socially situated. These insights suggest the utility of flow theory and PAT model in explaining not only productive engagement but also problematic technology use in education.

This study also offers actionable insights for educational practitioners and software developers. For educators, mitigating excessive translation software use, particularly among low-proficiency learners, requires balancing flow’s motivational benefits with critical engagement. Curricular design should integrate explicit guidance on ethical and effective use, complemented by structured activities such as post-editing and human-machine comparison tasks. These tasks encourage reflective evaluation of translation quality, enabling learners to experience flow through skill-appropriate engagement without fostering uncritical dependence. Task design should also consider proficiency differences, as lower-proficiency learners are more vulnerable to tool-driven flow that substitutes for deeper cognitive processing. To address this, scaffolded, projected-based tasks with calibrated difficulty can channel flow into constructive learning while discouraging overuse.

For software developers, the findings indicate that perceived translation quality shapes students’ flow, which then influences usage patterns. Enhancing algorithmic accuracy, contextual sensitivity and domain-specific vocabulary databases may strengthen users’ enjoyment and sense of control, while reducing reliance driven by repeated errors. Additionally, recognizing the role of social norms in collectivistic learning environments, developers could embed prompts that encourage ethical use or teacher-endorsed guidelines within the interface. Such features would reposition translation software from being a passive linguistic shortcut to becoming an interactive, pedagogically aligned tool that facilitates balanced human-machine collaboration.

### 5.4 Limitations

This study presents certain limitations that warrant consideration for the interpretation and applicability of our results.

Firstly, as an exploratory study investigating excessive use and flow dynamics in translation technology, the stratified convenience sampling approach inherently prioritizes contextual richness over population representativeness. This is reflected in demographic imbalances that constrain generalizability but enable preliminary pattern detection. Findings remain culturally situated within Chinese undergraduates’ linguistic and social norms, warranting verification through probability sampling across diverse learner populations.

Secondly, this study focuses solely on the relationship between flow and excessive use, resulting in limited explanatory power due to omitting other influential factors. Future research is therefore encouraged to integrate variables such as learner motivation and technology self-efficacy into the analytical framework to enhance the model’s explanatory power regarding excessive use.

Another limitation is that despite its theoretical grounding, the expected link between task perception, which captures challenge-skill balance, and flow was not observed in this study. Future research should explore this discrepancy either by examining whether the tool’s mediation weakens learners’ need to actively align skills with challenges, or by refining measures of task perception to reflect technology-driven shifts in EFL contexts.

Furthermore, the cross-sectional design of this study limits the establishment of definite causal links, and the observed associations thus cannot be construed as causal evidence. Future research is therefore suggested to adopt prospective designs to track variables over time and affirm the causal relationships among the key constructs.

## 6. Conclusion

To investigate whether EFL learners excessively use translation software and clarify the mechanisms underlying such behavior, this study applies flow theory and the person-artefact-task (PAT) model to analyze flow, its antecedents, and boundary conditions. Findings show excessive use among sampled Chinese undergraduates, with flow significantly predicting such behavior, especially among low-proficiency learners like non-English majors. Among factors influencing flow, social norms stand out as the most prominent, alongside perceived translation quality and task-technology fit, which collectively shape learners’ immersive engagement with translation tools. However, task perception shows no significant effect, likely because students’ trust in the tool’s quality diminishes the influence of task-related perceptions on flow.

The findings empirically confirm EFL learners’ over-reliance on translation tools [5,9,11]. They show flow, driven by social norms, perceived translation quality, and task-technology fit, predicts excessive use, with effect moderated by language proficiency. This extends flow theory to translation technology-assisted EFL learning contexts, and clarifies how positive experience interacts with individual attributes to foster excessive usage in educational settings. Practically, the results suggest strategies to promote critical, adaptive tool use in pedagogy, offering evidence-based guidance for balancing technological engagement with effective learning practices and supporting translation software’s integration into language education.

## Supporting information

S1 Dataset Survey data. (XLSX)

S1 Table Construct measurement and sources. (PDF)
