# Supplementary material for: Understanding EFL learners’ excessive use of translation software: An extension of the flow theory
Source: PLoS One. 2025 Nov 14;20(11):e0335610. doi: 10.1371/journal.pone.0335610 (PMC12617918; doi:10.1371/journal.pone.0335610)
Supplement: S1 Table — (PDF) [file pone.0335610.s002.pdf]

Construct measurement and sources.

Construct	Item	Measurement	Source
Perceived translation quality (PTQ)	PTQ1	The translated texts produced by the translation software are accurate in terms of meaning.	Liu et al. (2022)
	PTQ2	The translated texts produced by the translation software are accurate in terms of sentence structures.	
	PTQ3	The translated texts produced by the translation software are accurate in terms of style.	
	PTQ4	The translated texts produced by the translation software are accurate in terms of idiomatic expressions.	
Task perception (TP)	TP1	I am challenged by the translation tasks, but I believe my skills allow me to meet the challenges in the tasks.	Guo et al. (2015)
	TP2	My abilities match the high challenge of the translation tasks.	
	TP3	I feel I am competent enough to meet the demands of the translation tasks.	
Task-technology fit (TTF)	TTF1	Translation software is fit for the requirements of my translation tasks.	Wu & Chen (2017)
	TTF2	Translation software fits with my translation practice.	
	TTF3	When fulfilling translation tasks, I find it's easy to understand which function to use in translation software.	
	TTF4	Translation software functions are suitable for helping me with translation tasks.	
Social norms (SN)	SN1	My teachers think that using translation software is good for fulfilling translation tasks.	Strzelecki & ElArabawy (2024)
	SN2	People I know influence me to try out translation software for fulfilling translation tasks.	
	SN3	I read/see media message that using translation software is a good way of fulfilling translation tasks.	
Flow (FL)	FL1	When working on translation tasks with translation software, I am immersed in the task.	Cheng (2021)
	FL2	I enjoy the pleasure of using translation software to complete translation tasks.	
	FL3	It's exciting for me to use translation software to complete translation tasks.	
	FL4	I feel I have perceived control when I am using translation software in translation.	Webster et al. (1993)
Excessive use (EU)	EU1	I have made unsuccessful attempts to reduce the time using translation software to fulfill translation tasks.	Chen et al. (2017)
	EU2	I find it difficult to cut down the time I use translation software to fulfill translation tasks.	
	EU3	When doing translation, I have a hard time trying to resist the urge to use translation software.	
	EU4	When not using translation software, I have a hard time fulfilling translation tasks.	Hipp et al. (2023)
